# Microfabricated rGO/PANI Interdigitated Electrodes for Reference-Free, Label-Free pH Sensing on Flexible Substrates

**DOI:** 10.3390/mi16121337

**Published:** 2025-11-27

**Authors:** Maryam Sepehri Gohar, Ekin Asim Ozek, Melih Can Tasdelen, Burcu Arman Kuzubasoglu, Yaser Vaheb, Murat Kaya Yapici

**Affiliations:** 1 Faculty of Engineering and Natural Sciences, Sabanci University, 34956 Istanbul, Türkiye; sepehrigohar@sabanciuniv.edu (M.S.G.); mctasdelen@sabanciuniv.edu (M.C.T.); burcu.arman@sabanciuniv.edu (B.A.K.); 2 Micro/Nano Devices and Systems Lab (SU-MEMS), Sabanci University, 34956 Istanbul, Türkiye; ekinozek@sabanciuniv.edu (E.A.O.); yvaheb@yahoo.com (Y.V.); 3 Laboratory for Bio- and Nano-Instrumentation, École Polytechnique Fédérale de Lausanne (EPFL), 2002 Neuchâtel, Switzerland; 4 Department of Electrical and Computer Engineering, University of Washington, Seattle, WA 98195, USA; 5 Sabanci University Nanotechnology Research and Application Center (SUNUM), 34956 Istanbul, Türkiye

**Keywords:** flexible pH sensor, reduced graphene oxide (rGO), polyaniline (PANI), rGO/PANI composite, interdigitated electrodes (IDEs), photolithography, oxygen plasma etching, dip-coating, PET substrate

## Abstract

We present a flexible pH sensor which leverages the unique properties of reduced graphene oxide/polyaniline (rGO/PANI) composite films through an efficient and scalable hybrid microfabrication approach, wherein the rGO/PANI films are conformally coated on flexible polyethylene terephthalate (PET) substrates via dip-coating and thereafter lithographically patterned into precise arrays of interdigitated electrodes (IDEs), serving both as the pH-active medium and the electrical interface. Upon dip-coating, a thermal reduction process is performed to yield uniform rGO/PANI composite layers on PET substrates, where the PANI content is adjusted to 20% to optimize conductivity and protonation-driven response. Composition optimization is first performed using inkjet-printed silver (Ag) contacts and a conductometric readout mechanism is employed to explore pH-dependent behavior. Subsequently, IDE arrays are defined in the rGO/PANI using photolithography and oxygen-plasma etching, demonstrating clean pattern transfer and dimensional control on flexible substrates. Eliminating separate contact metals in the final design simplifies the stack and reduces cost. A set of IDE geometries is evaluated through I–V measurements in buffers of different pH values, revealing a consistent, monotonic change in electrical characteristics with pH and geometry-tunable response. The present study demonstrated that the most precise pH measurement was achieved with an 80:20 rGO/PANI composition within the pH 2–10 range. These results establish rGO/PANI IDEs as a scalable route to low-cost, miniaturized, and mechanically compliant pH sensors for field and in-line monitoring applications.

## 1. Introduction

Monitoring pH in situ is crucial across biomedicine, food processing, environmental analysis, and wearable health tracking; nevertheless, conventional glass pH electrodes are bulky, fragile, and poorly suited to conformal applications [1]. Recent advances in carbon-based flexible electronics have enabled thin, skin-compatible pH sensors with rapid responses and low operating voltages [2]. Graphene, owing to its high carrier mobility and large interfacial area, has emerged as a promising platform for miniaturized electrochemical transducers and can be directly patterned on polymer foils for scalable, wearable manufacturing [3]. Recent reviews cover both implantable electrochemical microsensors for in vivo monitoring, highlighting constraints on size, drift, and biocompatibility, as well as wearable sweat platforms where pH is a core target for real-time assessment [4,5].

Among proton-active polymers, polyaniline (PANI) is attractive because emeraldine base/salt (EB/ES) interconversion predictably modulates interfacial potential and conductivity in a pH-dependent manner. In addition to lithographic routes, printed PANI composites on flexible substrates offer protonation-coupled transduction with on-body form factors [6]. For instance, fully screen-printed PANI composites on textiles have delivered robust potentiometric pH readouts in flexible formats [7]. When PANI is combined with carbonaceous materials, PANI’s fast proton-coupled redox couples to the low-impedance electron transport of the carbon phase, typically boosting sensitivity, shortening response times, and improving mechanical stability on flexible substrates. Illustrative cases include PANI on Polyethylene terephthalate (PET) for sweat pH monitoring [8], hydrodynamics-engineered PANI/graphene films with Nernstian sensitivity [9], and wafer-scale PANI-grafted reduced graphene oxide (rGO) channels that reduce hysteresis [10]. Beyond printed and rGO-modified films, layer-by-layer graphene oxide (GO)/PANI architecture enables stable thin-film potentiometric operation [11] and extended-gate graphene transducers have demonstrated low drift, suitable for long-term monitoring [12]. PANI–carbon nanotube (CNT) nanoyarns further show coaxial architectures for textile pH sensing [13].

At the material level, graphene’s apparent pH sensitivity is strongly modulated by defect density and surface chemistry, shifting from interfacial electrostatic gating on low-defect graphene to protonation-driven interactions as disorder/functionalization increase [14,15]. These insights guide carbon/PANI choices for rGO, the reduction state and defect density (sp^2^/sp^3^ balance, oxygenated groups) govern charge transfer and double-layer coupling relevant to pH transduction [16]; within PANI, acidification drives ES/EB transitions and polaron/bipolaron formation, increasing film conductivity [17,18]. Embedding PANI through an rGO percolation network couples chemical gain to a low-impedance electronic path, an effect widely reported for graphene/PANI hybrids [19]. An optimum rGO:PANI composition is therefore expected, since increasing PANI strengthens proton-coupled modulation but can raise series resistance and weaken lithographic robustness, whereas higher rGO preserves conduction and patternability; these trade-offs align with established GO to rGO reduction chemistry and its impact on transport and interfacial interactions [20,21].

Conductometric (chemiresistive) films patterned as interdigitated electrodes (IDEs) provide a simple, reference-free I–V readout of pH-dependent conductance compatible with flexible substrates [22]. In such two-terminal devices, a small bias probes net conductance across the interdigitated gap; pH modulates the electric double layer and the protonation state of PANI (and surface charge at rGO defect/oxygen sites), yielding a reproducible conductance change without a reference electrode. This mechanism is consistent with impedance-based, reference-electrode-free pH sensing on oxide electrodes, where low-frequency double-layer capacitance varies with surface protonation [23]. Interdigitated arrays are well-established geometries for two-terminal electrochemical transduction [24], and recent flexible IDEs pH platforms further underscore the practicality of reference-free architectures for field use [25]. Finally, reports of super-Nernstian behavior typically arise in titration/voltammetric modes rather than equilibrium potentiometry; distinguishing transduction mode, geometry, and operating bias is essential for reproducibility [26,27].

**Figure 1:**
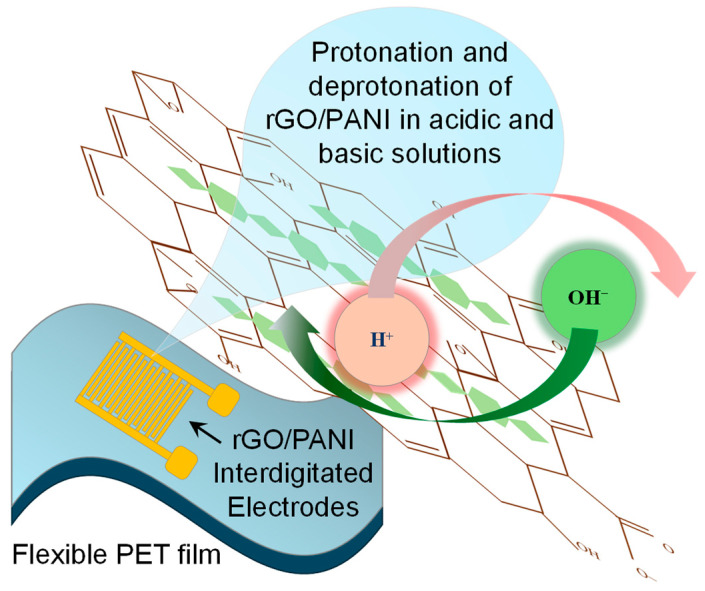
Mechanism of pH-dependent protonation and deprotonation of rGO/PANI IDEs under acidic and alkaline electrolyte conditions.

Here, we introduce a miniaturized and reference-free pH sensor on PET in which an rGO/PANI composite is photolithographically patterned into IDEs that simultaneously serve as the electrodes and the sensing material, enabling I–V readout with no additional coating. The sensing mechanism is based on the coupled pH-dependent protonation/deprotonation of PANI nitrogen sites and ionization of oxygen-containing groups on rGO, which modulate H^+^/OH^−^ adsorption at the composite/electrolyte interface and the polaron density along the conjugated backbone, thereby translating local pH changes into reproducible conductance variations across the IDE channel (Figure 1). To our knowledge, this work presents the first single-material rGO/PANI IDE architecture on a flexible substrate. We systematically vary IDE geometry and optimize the rGO:PANI ratio to define a simple, manufacturable design space that yields reliable pH discrimination under low bias and is compatible with flexible operation.

## 2. Materials and Methods

### 2.1. Reagents and Substrates

Aniline, hydrochloric acid (HCl, 1 M), ammonium peroxydisulfate (APS), graphene oxide (GO) suspension (4 mg mL^−1^), PET films, AZ5214E photoresist (Microchemicals GmbH, Ulm, Germany), AZ 726 MIF developer (Merck Performance Materials GmbH, Wiesbaden, Germany), and JS-A191 silver inkjet ink (NovaCentrix, Austin, TX, USA) nanoparticle (AgNP) were used as received.

### 2.2. PANI Synthesis, rGO/PANI Composite Preparation, and PET Coating

**Figure 2:**
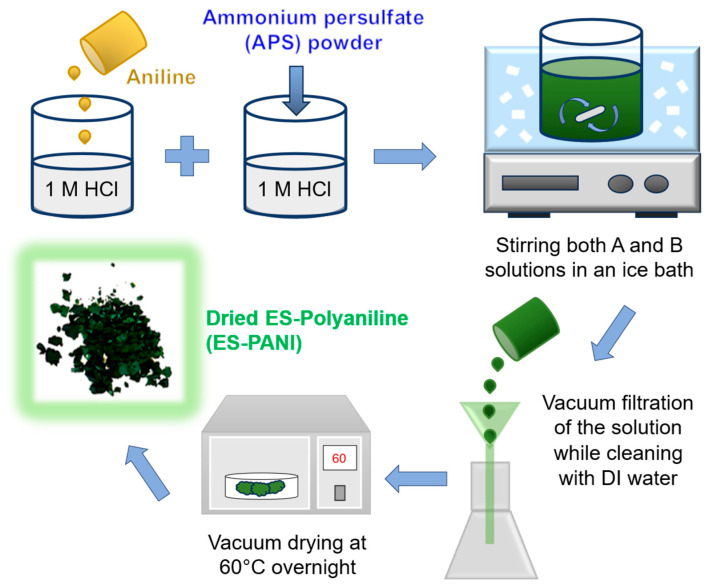
Schematic description of PANI synthesis by chemical oxidative polymerization.

PANI powder was synthesized through oxidative polymerization in an acidic environment. Two precursor solutions were prepared in 1 M HCl: Solution A consists of aniline (6 mL) dissolved in 75 mL of hydrochloric acid; Solution B comprises ammonium persulfate (7.5 g) in 75 mL of hydrochloric acid. Solutions A and B were combined and stirred for 3 h in an ice bath, then removed from the bath, and then stirred a further 5 h at room temperature to complete polymerization. The precipitate was rinsed with deionized (DI) water and dried overnight at 60 °C, producing the dark-green ES form of PANI. The dried PANI was redispersed in DI water by probe sonication to prepare a 4 mg mL^−1^ PANI dispersion (Figure 2).

A commercial GO dispersion (as received, 4 mg mL^−1^ in water) was used. GO:PANI ratios (95:5, 90:10, 85:15, 80:20) refer to mass fractions (*w*/*w*) of solids in the coating dispersion. Since both GO and PANI stock dispersions were 4 mg mL^−1^, the target mass fractions were obtained by simple volumetric mixing (e.g., 80:20 *w*/*w* = 4:1 *v*/*v*). The resulting co-dispersions were probe-sonicated to homogenize prior to dip-coating.

Coating parameters and the subsequent thermal reduction followed our previously reported protocol [28]. Dip-coating was performed manually with an immersion time of 30 s, a steady (unmetered) withdrawal, and four coating cycles. After each withdrawal, the film was air-dried for ~60 s and then baked for 10 min at 60 °C. Because the withdrawal speed was unmetered, process control was implemented via the cycle count. This 4-cycle recipe yielded continuous films that withstood photolithography and O_2_ plasma etching and exhibited stable electrical behavior. Detailed parameters for dispersion preparation, dip-coating, and reduction are summarized in Table 1, including target rGO:PANI mass ratio, immersion time, withdrawal mode, number of cycles, inter-cycle drying/bake, and reduction conditions.

### 2.3. Screening Layout of İnkjet-Printed AgNP Bars

**Figure 3:**
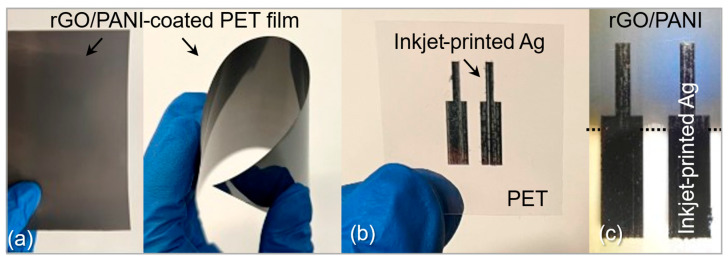
(**a**) The rGO/PANI/PET films with different ratios of the material, (**b**) Inkjet-printed silver electrodes on PET, (**c**) Inkjet-printed silver electrodes on rGO/PANI-coated PET films.

Prior to microfabrication, a screening structure (Figure 3) was fabricated to compare pH response of films prepared from different GO:PANI ratios by investigating the potentiometric measurements. Silver bars (6000 µm × 1250 µm, 4100 µm gap) were inkjet-printed on the coated PET (Fujifilm Dimatix DMP-2850, Santa Clara, CA, USA) using the JS-A191 AgNP ink and cured at 150 °C for 30 min. To protect Ag during brief immersions in pH 2–10 buffers, the PR is applied on top of Ag bars using a temporary image-reversal process including the following steps: soft-bake at 110 °C for 2 min, UV exposure for 5 s using a Midas mask aligner, hard-bake at 145 °C for 15 min, and a second UV exposure for 15 s. This image-reversal step hardens the PR and improves its short-term resistance to aqueous acid/base exposure and is commonly used in alkaline developers. Importantly, the PR is absent from the final rGO/PANI IDE sensors, which contain no Ag and are measured without any overcoat. During the brief screening used to select the most sensitive ratio, Ag edges remained intact and bar conductivity was stable. Any PR attack or Ag degradation would have manifested as drift or undercut; neither was observed under the test conditions. Although a 75:25 rGO:PANI composition was evaluated, it proved incompatible with the lithography/development sequence (partial lift-off; see Section 2.4). Accordingly, we selected rGO:PANI = 80:20, which met microfabrication requirements; screening also identified 80:20 as the most pH-sensitive composition, and it was used for IDE fabrication.

### 2.4. Selection of Optimal rGO/PANI Ratio Concentration for Microfabrication

Using a GO/PANI co-dispersion, rather than neat PANI; GO (later rGO) provides film formation and adhesion on PET, while PANI remains embedded and proton active. During process qualification, PET-supported rGO/PANI coatings spanning rGO mass fractions of 100, 95, 90, 85, and 80% withstood the photolithography and plasma-etch sequence under identical conditions. In contrast, the 75:25 composition partially lifted during development and was therefore not investigated further for device fabrication. We attribute this to the combination of (i) lower rGO content, which diminishes the hydrophobic, mechanically stable scaffold on PET, and (ii) the strong alkaline Tetramethylammonium hydroxide (TMAH) developer, which deprotonates PANI and deprotonates oxygenated sites on rGO, increasing film hydrophilicity and weakening interfacial adhesion during the wet step. These observations indicate that compositions that are optimal for film formation are not necessarily robust to microfabrication chemistry, and that developer strength, PR system, and exposure/bake conditions must be tuned with composition [29].

### 2.5. Interdigitated Electrode (IDE) Design and Microfabrication of rGO/PANI

**Figure 4:**
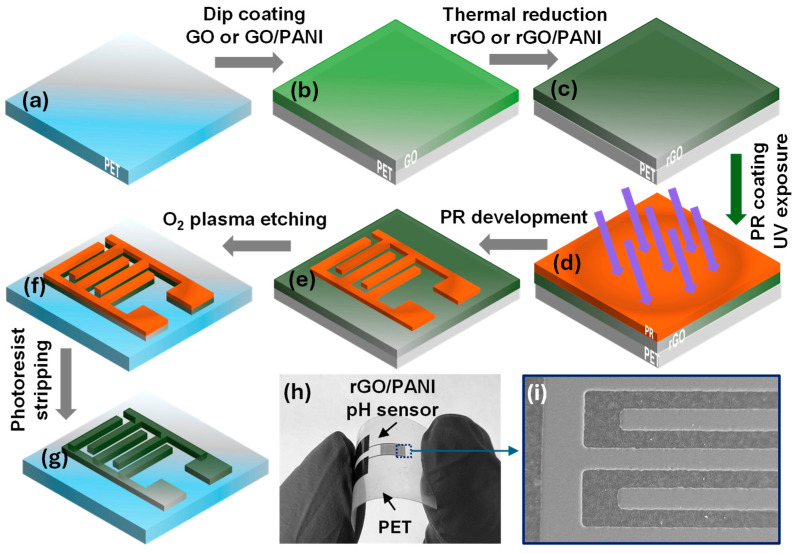
Developed fabrication technology process schematic flow for rGO, and rGO/PANI-clad flexible PET films: (**a**) preparation of PET substrate, (**b**) dip-coating of PET in GO or GO/PANI, (**c**) thermal reduction of GO or GO/PANI-clad PET flexible films to rGO or rGO/PANI, (**d**) photoresist (PR) spinning and UV-exposure photolithography of conductive flexible films, (**e**) PR development, (**f**) oxygen plasma etching of rGO or rGO/PANI films, (**g**) PR stripping, (**h**) fabricated interdigitated rGO/PANI pH sensor on a flexible PET substrate, (**i**) SEM image of the fabricated sensor.

IDE structures were patterned directly on the conductive optimal rGO/PANI (80:20) films using standard photolithography after coating and thermal reduction (Figure 4a–g). A 1.6 µm photoresist (AZ5214E) layer was spin-coated (G3P-8, Specialty Coating Systems Inc., Indianapolis, IN, USA) at 2000 rpm for 30 s, exposed to ultraviolet light by a mask aligner (MDA-60MS, MIDAS SYSTEM Co., Ltd., Daejeon, Republic of Korea) for 10 s (Figure 4d). For uniform feature transfer on flexible PET, the PET mask and substrate were pressed between two flat glass plates during exposure (glass-sandwich configuration). Then the development was carried out in AZ 726 MIF (TMHA) for 90 s to create the IDEs, and an immediate DI water rinse followed by N_2_ dry. Unprotected film was removed by oxygen plasma etching (50 W, 10 min) using a Plasmalab System 100 ICP 300 DRIE device (Oxford Instruments, Yatton, UK). The photoresist (PR) was stripped in acetone (ISOLAB Laborgeräte GmbH, Eschau, Germany) at room temperature for ≤5 s (Figure 4g), followed by IPA (ISOLAB Laborgeräte GmbH, Eschau, Germany) rinse and N_2_ dry; no ultrasonics were used to protect the rGO/PANI edges and PET. More detailed parameters of the lithography process are mentioned in Table 2. A representative SEM (Sigma FE-SEM (Carl Zeiss Microscopy GmbH, Jena, Germany) in Figure 4i shows clean removal of the sensitive film between digits with preservation of IDE fidelity.

**Figure 5:**
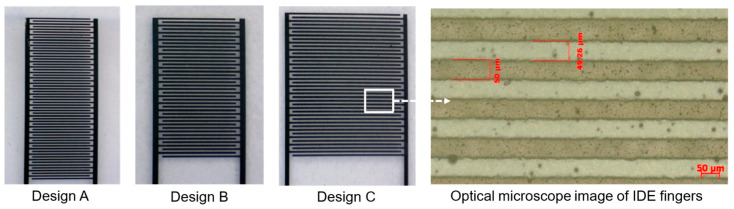
The image of three IDEs designs and the optical microscope image of the IDE fingers.

To isolate the effects of finger count and length while maintaining finger width and inter-finger gap constant (Table 3), three geometries were fabricated as shown in Figure 5.

## 3. Results and Discussion

### 3.1. Characterization

**Figure 6:**
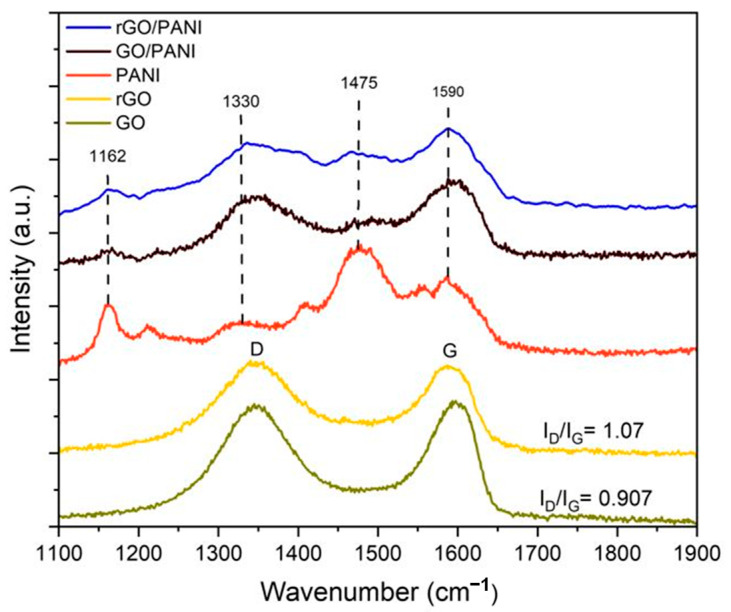
Raman spectra of GO, rGO, PANI, GO/PANI.

GO, rGO, GO/PANI, and rGO/PANI-coated PET films were examined by Raman spectroscopy (inVia Reflex Raman microscope and spectrometer, 532 nm, Renishaw plc, New Mills, Wotton-under-Edge, Gloucestershire, UK) to evaluate the degree of reduction and the effect of PANI (Figure 6). Across samples, the D and G bands were observed in the range of 1308–1375 cm^−1^ and 1536–1599 cm^−1^, respectively. We monitored the intensity ratio I_D_/I_G_ as a measure of reduction; GO displayed a ratio of 0.907, which increased to 1.07 following low-temperature reduction, confirming the production of rGO on PET. Introducing PANI attenuated the apparent reduction signature: the GO/PANI film showed I_D_/I_G_ = 0.931, and rGO/PANI decreased from the rGO value to 0.953. Nonetheless, the I_D_/I_G_ of rGO/PANI remained higher than GO/PANI (0.931), indicating that the rGO character is predominantly preserved inside the composite. PANI regulates the effective reduction, hence influencing the Raman-derived disorder/graphitic domain equilibrium, while the rGO component remains observable in the rGO/PANI composite. In addition to the carbon framework peaks, rGO/PANI displayed distinctive PANI bands at approximately 1590, 1475, 1330, and 1162 cm^−1^, assigned to C=C (quinoid) stretching, C=N stretching, C–N^+^ stretching, and C–H bending in the benzenoid ring, respectively [30]. Pure PANI and GO/PANI exhibited comparable PANI signatures owing to the identical polyaniline backbone. The persistence of PANI signatures after reduction and through lithographic processing indicates that PANI is retained within the rGO network on PET.

### 3.2. Potentiometric Behavior of the Composite

**Figure 7:**
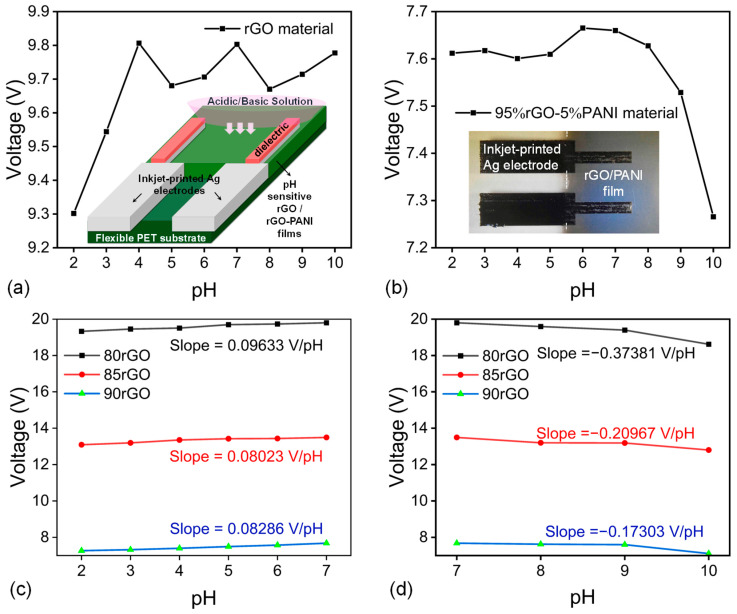
(**a**,**b**) Comparison of the potentiometric pH response of pure rGO and a 95:5 rGO:PANI composite using the device configuration shown in Figure 3 and in the plot insets, where the sensing films are coated on PET and contacted by inkjet-printed Ag bar electrodes on top of the coated layer. (**c**,**d**) Potentiometric pH response of 80:20, 85:15, and 90:10 rGO:PANI composites in the acidic and alkaline regions using the same PET-based architecture with inkjet-printed Ag electrodes.

Initially, we compared the response of pure rGO-coated PET film against that of a 95:5 (rGO:PANI) composite film as sensing layers for pH. For these potentiometric measurements, we used the device configuration depicted in Figure 3, where the rGO or rGO:PANI films were coated onto PET substrates and contacted by Ag bar electrodes on top of the coated layer. A dielectric layer (i.e., photoresist) was applied on top of the bar electrodes to insulate the silver electrodes when subjected to various acidic/basic solutions. Next, the pH sensitive rGO:PANI area was immersed in various solutions with pH in the range of 2–10, while a constant 1µA current was applied. It took approximately 80 s for the voltage to stabilize and produce reliable data. The resulting voltage values were sampled a hundred times, and their average was plotted (Figure 7a,b).

As evident from Figure 7a, pure rGO responded to pH but displayed weaker stability and lacked a monotonic trend across the acidic and alkaline range. The incorporation of even 5% PANI stabilized the response and improved linearity, underscoring PANI’s function in providing a more consistent protonation/deprotonation mechanism within the percolated rGO network.

### 3.3. Composition Optimization

Subsequently, we evaluated rGO:PANI ratios of 90:10, 85:15, and 80:20 (Figure 7c,d). The 90:10 ratio had a superior reaction in strong acid compared to the 85:15 ratio, although the difference was minimal. The 80:20 composite exhibited the highest sensitivity in both acidic and alkaline conditions, leading to its selection for device-level testing.

### 3.4. Performance of rGO/PANI Based pH Sensor

**Figure 8:**
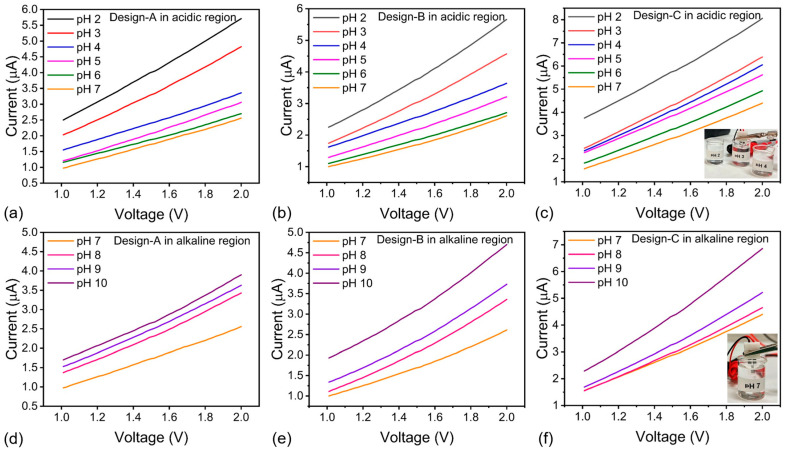
(**a**–**f**): I–V response of rGO/PANI-based IDE-type pH sensor designs (design A, B, and C) when subjected to acidic and alkaline media as shown in the plot insets.

Using a Keysight B2902B source-measure unit on a probe station, we recorded I–V curves of IDEs coated with the optimized 80:20 rGO/PANI film in standard buffers (pH 2–10). Buffers were prepared by titrating distilled water with 0.1 M hydrochloric acid and 0.1 M potassium hydroxide. For acidic solutions, proton absorption at the rGO/PANI/electrolyte interface increases the proportion of protonated ES, leading to higher conductivity and hence larger currents (Figure 8a–f). At higher pH, OH^−^ neutralizes the doped PANI, driving the ES to EB transition and lowering conductivity; accordingly, currents decrease in alkaline medium. The difference in current between acidic setpoints exceeds that in alkaline setpoints, consistent with the higher mobility and faster interfacial kinetics of H^+^ relative to OH^−^. These observations collectively indicate that PANI doping provides the rGO network with a robust, reversible acid–base transduction mechanism while preserving sufficient graphitic ways for charge transport.

**Figure 9:**
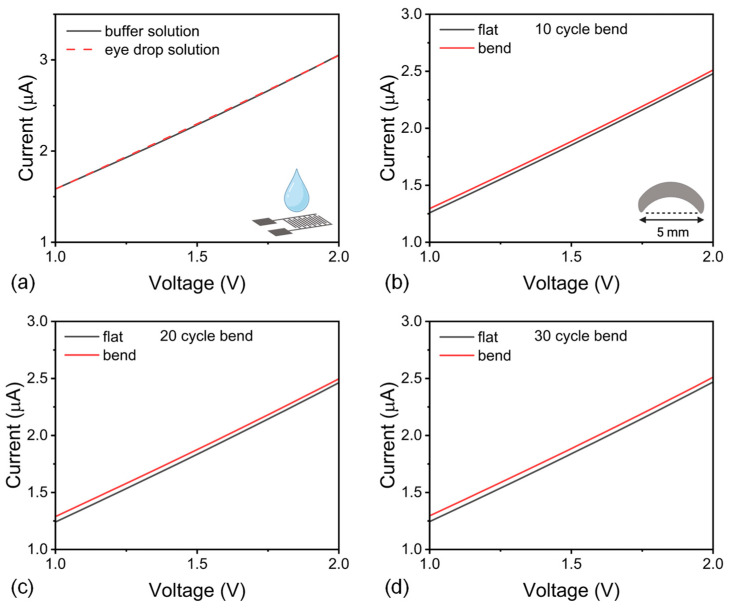
(**a**) I–V curves at pH = 6.2: eye drop versus prepared buffer solution at the same pH value, (**b**) Evolution of I–V characteristics under cyclic bending (flat vs. bent) for design A in pH 7 solution after 10 cycles, (**c**) After 20 cycles, (**d**) After 30 cycles.

Extending the analysis beyond simple buffers, a commercial eye drop was utilized for the purpose of incorporating an application-matrix analysis. In a complex matrix (commercial eye drops; FULLFRESH^®^ 5.60 mg/2.40 mg single-dose, Vem Pharmaceuticals Industry and Trade Inc., Tekirdag, Türkiye), the I–V characteristic of pH=6.2 was retained, with only a small offset versus the KOH buffer with the same pH value as shown in Figure 9a; at +2.0 V, I(buffer)=305.314 μA and I (eye-drop)=304.894 μA (∆I=0.42 μA, 0.14%), corresponding to $∆\ln I=1.38\times 10^{-3}\;\mu A$. Thus, excipients introduced a minor, readily correctable matrix effect while preserving pH discrimination.

Additionally, as a practical check of device robustness, we next examined I–V behavior under inner-arc bending. The bending was imposed using a 5 mm-diameter cylindrical support (R=2.5 mm). The outer-surface strain was estimated as ε≈t/2R; where *t* is the substrate thickness and R is the bending radius. For the PET substrate (t≈0.2 mm), ε≈4%. The IDE active region of design A was placed on the inner arc (compression). I–V characteristics were recorded in the flat state (Figure 9b–d), after ten preconditioning bend cycles, and while bent at R=2.5 mm, using the same solution as in static tests at pH=7; measurements were performed in triplicate (n=3). For quantitative comparison, Table 4 lists the absolute currents for flat and bent states and the percent change at a 2 V bias in a pH=7 solution. Under compression, the current increased slightly, consistent with contact tightening within the rGO/PANI network.

**Figure 10:**
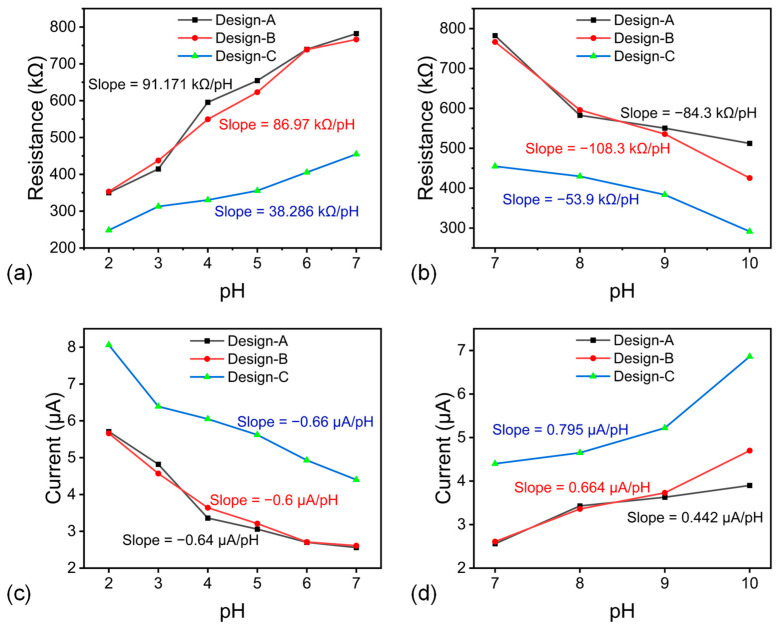
(**a**,**b**) The resistance values correspond to the 2 V voltage value in acidic and alkaline medium, (**c**,**d**) The current values correspond to the 2 V voltage value in acidic and alkaline medium.

The effect of IDE designs differing in finger count and finger length (designs A–C) was evaluated. For each device, resistance and current values were extracted at a fixed 2 V bias from the I–V curves and plotted versus pH values as shown in Figure 10a–d. In acidic solutions, designs A and B, differing mainly in finger quantity, exhibited similar slopes, indicating that within our dimensional range, the finger count has a second-order effect on pH sensitivity. In contrast, design C, with a 4 mm finger length, produced a noticeably higher current (lower resistance), reflecting the larger active interfacial area and longer overlap length between electrodes. Thus, extending finger length is more effective than increasing finger number for enhancing the transduction signal under otherwise identical film and electrolyte conditions.

As a scalability metric, film uniformity was assessed by four-point-probe sheet-resistance mapping (Cascade Microtech CP4, Cascade Microtech Inc., Beaverton, OR, USA), which showed consistent rGO/PANI coverage across the active area (representative $R_{s}$ = 35.6 Ω/sq on 5 cm PET after reduction). All processing was performed at moderate temperatures (≤180 °C) using commercially available materials and polymer substrates. The device architecture was defined by laboratory-scale photolithography, and the layer stack and process parameters are compatible with roll-to-roll manufacturing, including printing workflows.

## 4. Conclusions

We demonstrated flexible, PET-based micro pH sensors employing rGO/PANI thin films formed by low-temperature processing. Raman analysis verified successful reduction of GO to rGO on PET and confirmed characteristic PANI vibrations; while PANI slightly moderated the Raman I_D_/I_G_ signature of reduction, it enhanced electrochemical transduction. Electrical measurements across pH 2–10 identified an 80:20 rGO/PANI composition as optimal, yielding stable responses and improved linearity, particularly under conditions, through the reversible protonation/deprotonation of the PANI phase within the rGO percolation network. Device-level comparisons further showed that increasing IDE finger length (rather than finger count) more effectively amplifies signal magnitude, consistent with a larger active interfacial area.

These results indicate that composition (rGO:PANI ratio) and microelectrode geometry are complementary levers for sensitivity and stability in flexible potentiometric sensors. The dip-coating/low-temperature route is compatible with scalable, large-area fabrication on polymer substrates, supporting integration into lightweight, conformable platforms. Future work will target long-term drift and hysteresis, ion selectivity and interference, mechanical durability under bending, and validation in actual samples to advance the sensors for practical field and wearable applications. Additionally, the compatibility of inkjet printing with roll-to-roll manufacturing will be investigated to further develop the sensors for practical field and wearable applications.

## Figures and Tables

**Table 1:** Process parameters for dispersion and dip-coating.

Stage	Parameter	Value/Setting	Rationale/Note
Dispersion	GO dispersion	4 mg mL^−1^ in deionized water	Provides a highly stable colloidal dispersion and minimizes batch-to-batch concentration deviation.
	PANI dispersion	4 mg mL^−1^ in deionized water	Adjusted to enable volumetric mixing toward the targeted rGO:PANI mass ratio.
	Target composition	80:20 (rGO:PANI, *w*/*w*)	Identified as the optimum composition balancing pH-sensing performance and patternability.
Composite homogenization	Mixing protocol	Volumetric mixing of rGO and PANI dispersions to reach the desired *w*/*w* ratio	Ensures accurate control over composite composition.
	Homogenization	Probe sonication	Promotes a uniform rGO-PANI composite and suppresses agglomeration.
Dip-coating Deposition	Immersion time	30 s per cycle	Provides practical control over deposited film for manual processing.
	Withdrawal	Manual withdrawal at a steady pace	Minimizes edge defects.
	Number of cycles	4 dip-coating cycles (single face of the substrate)	A single cycle yields continuous but high-resistance films; 4 cycles yield lower sheet resistance and films robust to subsequent lithographic steps.
Thermal Treatments	Intermediate drying	60 °C for 10 min in an oven (after each deposition cycle)	Improves film continuity, promotes solvent removal, and reduces run-off during successive cycles.
	Reduction	180 °C for 3 h	Ensuring the conversion of GO to rGO at a temperature that PET can withstand.
Film verification	Structural characterization	Raman spectroscopy	Confirms the presence and integrity of rGO and PANI components in the composite.
	Electrical characterization	Sheet resistance ≈ 35.57 Ω sq^−1^	Demonstrates reduction and functional conductivity suitable for device integration.

**Table 2:** Parameters of the lithography process of IDEs.

Step	Purpose	Material/Tool	Nominal Setting	Notes/Range
1. PR coat	IDE pattern	AZ5214E; spin coater	2000 rpm, 30 s	Thickness ~1.6 µm; 25 °C
2. UV exposure	Pattern PR	Mask + substrate between two flat glass plates; Mask aligner	10 s UV exposure	Ensures uniform contact on flexible PET
3. Develop	Clear exposed PR	AZ 726 MIF	90 s, DI rinse	—
4. Rinse and dry	remove residues	DI water; N_2_ gun	5–10 s DI; N_2_ dry	—
5. O_2_ plasma etch	Remove unprotected rGO/PANI	DRIE device	50 W, 10 min	—
6. PR strip	Remove PR mask	Acetone, IPA	≤5 s acetone; then IPA	No ultrasonics
7. Visual check	Verify clearance	Optical/SEM	—	clean sidewalls

**Table 3:** Interdigitated electrode geometries used in this work. “Total fingers” denotes the sum over both interdigitated electrodes.

Geometry	Total Fingers	Finger Length (mm)	Finger Width and Finger Gap (µm)
Sensor A	60	2	50
Sensor B	40	2	50
Sensor C	40	4	50

**Table 4:** Current at a fixed bias (+2 V) for IDEs (design A) measured in pH 7 under flat and bent states (R = 2.5 mm, inner-arc compression) after 10, 20, and 30 bend cycles.

Bend Cycle	I_flat_ (μA)	I_bend_ (μA)	[(ΔI/I_flat_) × 100] (%)
10	2.47886	2.51094	1.29
20	2.46883	2.51094	1.69
30	2.46268	2.49681	1.37

## Data Availability

The original contributions presented in this study are included in the article. Further inquiries can be directed to the corresponding author.
